# How to Preserve Health

**Published:** 1890-09

**Authors:** 


HOW TO PRESERVE HEALTH. By Louis Barkan, M. D. New York: American News Co. 1890.

This is a handy little volume intended for the laity, and containing, as its name implies, numerous precepts for the care of the health.

The precepts are arranged under suitable headings that they may be readily found. It is, therefore, a useful book of reference. Thus we have an instructive chapter on food. Then we have a series of chapters on the hygiene of the different organs; hygiene of age and occupation, and hygiene of the dwelling. These constitute the first part.

The second part contains chapters on the care of the sick, nursing, the family physician, how to give aid in emergencies, contagious and miasmatic diseases; diseases of nervous system, the respiratory tract, the digestive tract, etc. The advice seems sound and simple and we can recommend it as a good book for every household. W. M. J.

